# The Impact of Persistent Irritability on the Medication Treatment of Paediatric Attention Deficit Hyperactivity Disorder

**DOI:** 10.3389/fpsyt.2021.699687

**Published:** 2021-07-21

**Authors:** Raman Baweja, Daniel A. Waschbusch, William E. Pelham, William E. Pelham, James G. Waxmonsky

**Affiliations:** ^1^Department of Psychiatry and Behavioral Health, Penn State College of Medicine, Hershey, PA, United States; ^2^Center of Human Development, University of California, San Diego, San Diego, CA, United States; ^3^Center for Children and Families Florida International University, Miami, FL, United States

**Keywords:** ADHD, CNS stimulant, irritability, medication, children

## Abstract

This study compares the efficacy and tolerability of central nervous system (CNS) stimulants in children with attention deficit hyperactivity disorder (ADHD) with and without prominent irritability (IRR) over the course of 30 months. This is a secondary analysis of a study examining growth patterns in medication naïve children with ADHD subsequently treated with CNS stimulants (predominantly OROS-Methylphenidate, up to 54 mg per day) for 30 months. Participants had to meet full diagnostic criteria for ADHD and been treated with CNS stimulants for under 30 days. Children were classified as IRR if they were rated as *pretty much* or *very much* on either of the “often angry” or easily annoyed” items plus “lose temper,” items of the Disruptive Behavior Disorders Rating Scale (DBDRS). Structured ratings of ADHD symptoms, impairment, side effects, and symptoms of oppositional defiant disorder (ODD) were collected every 2–12 weeks for the duration of the study. Medication use was measured by pill count and parent report. The IRR group comprised 28% of all participants. The IRR group had significantly higher levels of ADHD and ODD symptoms, impairment, and side effects ratings at baseline. In the IRR group, ODD symptoms, emotional lability, and impairment significantly decreased for participants with higher medication use. Total side effects increased for non-IRR participants with higher medication use. Emotional side effects decreased for IRR participants with higher medication use. Central nervous system stimulants were a tolerable and efficacious treatment in treatment naïve youth with ADHD with irritability.

**Clinical Trials Registration:** NCT01109849

## Introduction

Persistent non-episodic irritability is one of the most common presentations in child mental health (1) and presents in youth with a wide range of psychiatric diagnoses (2). Irritability, defined as proneness to anger, is commonly associated with attention deficit hyperactivity disorder (ADHD), as up to half of youth with ADHD have prominently elevated levels of irritability (3, 4). The presence of persistent irritability in children with ADHD increases the chance that they will present for treatment and is associated with increased impairment (5–8). Concerns over irritability and aggression are one of the main reasons for increasing dose of CNS stimulants as well as for polypharmacy in ADHD youth (9). Antidepressants, mood stabilizers, and atypical antipsychotics have all been prescribed to target these constructs (10–12). This prescription trend is concerning given the increased morbidity associated with these medications (13–15).

In the randomized 14-month treatment phase of the Multimodal Treatment Study of children with ADHD (MTA) study, Central Nervous System (CNS) stimulants were associated with large reductions in symptoms of ADHD and moderate reduction in levels of parent-rated irritability in participants with elevated irritability at baseline. Moreover, irritability did not appear to influence the treatment response for ADHD symptoms (6). However, the MTA is limited by the predominant use of immediate-release CNS stimulants as extended-release (ER) CNS stimulants are the current standard treatment (16). In addition, the MTA analysis assessed only the impact of irritability on the efficacy of CNS stimulants for reducing ADHD symptoms scores and irritability, using the same measure to both define irritability and measure treatment effects. It did not assess the impact of irritability on treatment tolerability or the change in impairment.

Outside of the MTA, most studies assessing the impact of CNS stimulants on irritability only briefly examined treatment effects before adjunctive medications were added. They enrolled participants meeting criteria for disruptive mood dysregulation disorder (DMDD)/severe mood dysregulation or recurrent physical aggression (8, 17–22). However, irritability that does not meet DMDD criteria or lead to persistent aggression can still be very impairing (23, 24). Hence, there is appreciable value in assessing the trajectory of irritability in children not meeting criteria for DMDD or manifesting prominent aggression.

The related concept of emotional lability refers to a more general dysregulation of mood. It is a commonly reported side effect of CNS stimulants that can lead to treatment discontinuation (25). The tolerability of CNS stimulants is typically assessed by spontaneous reports from parent and child. The few studies to systematically examine tolerability were of brief duration and produced mixed results in regards to the impact of baseline emotional symptoms (26, 27). Treatment naïve youth experience higher rates of adverse events then previously treated youth (28) making them the preferred population to assess the emotional tolerability of CNS stimulants. No prior work has examined the impact of efficacy and tolerability of chronic treatment with ER CNS stimulants in a large cohort of treatment naïve youth.

As part of a NIH-funded longitudinal study examining the impact of ER CNS stimulants on physical growth in medication naïve children with ADHD (29), we systematically examined the efficacy and tolerability of CNS stimulants in 230 children with ADHD with and without prominent irritability over the course of 30 months. It was hypothesized that: (a) prior to any use of CNS stimulants, children with high levels of irritability would have higher levels of ADHD symptoms, other behavior problems, physical and emotional symptoms often classified as side effects of CNS stimulants and impairment than children with low levels of irritability; (b) medication would be associated with sustained reductions in ADHD symptoms, and impairment, and (c) irritability would not moderate changes in symptoms, emotional lability, or other side effect levels with CNS stimulants.

## Methods

### Participants

Participants were drawn from a study that examined effects of stimulant medication on the physical growth of children with ADHD (29). Exclusion criteria for the original study were intelligence quotient <70, body mass index (BMI) below the 5^th^ percentile or above the 85^th^ percentile, use of other psychotropic medications, autism spectrum disorder, or medical contraindications to CNS stimulants. Of the 230 participants in the original sample, 226 were included in this study; the four children not included were missing irritability data at baseline. The participants were ages 5–12 years and met criteria for DSM-IV ADHD (any subtype) with a lifetime use of stimulant medication that was under 30 days. Only five participants (2.1%) had previously used any CNS stimulants.

Attention deficit hyperactivity disorder was diagnosed using the Disruptive Behavior Disorders Structured Interview (30), combined with parent and teacher ratings (31). Psychiatric comorbidity was assessed by the NIMH Diagnostic Interview Schedule for Children IV, computerized version (32), with comorbid diagnoses allowed if ADHD was the most impairing condition and they were not in need of other psychotropic medication besides CNS stimulants. Diagnoses were confirmed by two MD/Ph.D. faculty.

Following earlier research (33–35), participants were grouped based on the presence or absence of persistent irritability (IRR) as measured by the Disruptive Behavior Disorders Rating Scale (DBDRS). The DBDRS measures DSM symptoms of ADHD, ODD, and CD using a 0–3 Likert scale. Children were considered irritable if they were rated as 2 (*pretty much*) or 3 (*very much*) by parents on either of the “often angry” or “easily annoyed” items plus “lose temper,” which was also supplemented by Teacher-DBDRS. Others have used a similar symptom threshold to identify youth with behavioral problems as having elevated irritability or not (33, 34, 36). These 3 items also map directly on the DSM-5 ODD “angry/irritable mood” category (2) and the items in the Development and Well-Being Assessment, which has been used by others to assess irritability in pediatric populations (37, 38). Parent report on structured rating scales has been found to be as valid for measuring irritability in youth as clinician-administered interviews (39). This resulted in 63 of the 226 participants (27.9%) included in the IRR group and the remaining 163 (72.1%) were in the non-irritable (Non-IRR) group.

### Procedures

Participants were recruited through mailings to schools, primary care providers, and community mental health providers. As per protocol, families were randomly assigned to receive either medication or behavioral therapy in a 4 to 1 ratio (29). All procedures were approved by the Western Institutional Review Board. Written consent was obtained from parents and assent from children age seven or older. As part of the larger study, 50 were randomly assigned to behavioral treatments only and 180 to medication plus low dose behavioral treatments. All medication was prescribed through the study under open label conditions, as the primary outcome of the parent study was growth, which was objectively measured. Children in the medication arm were initially treated with OROS-Methylphenidate (MPH), starting at 18 mg with dose titrated every 2 weeks (maximum dose up to 54 mg per day) until optimized based on parent and teacher ratings. Optimal dose was defined, as a tolerable dose enabling participants to reach a level of home and school functioning considered good with no meaningful room for improvement with a tolerable level of side effects. This open-label trial could last up to 12 weeks. If OROS-MPH was not efficacious, swallowable, or tolerable, alternative MPH products or mixed amphetamine salts products were prescribed. Study treatment lasted up to 30 months with assessments at least every 3 months. Among the 226 participants, 161 (71.2%) used medication at some point during the study. Each measure was assessed at week 0 (baseline), week 2, 4, 6, 8, 10, 12, 16, 20, 24 then months 9, 12, 15, 18, 21, 24, 27, and 30. Additional assessments of ADHD symptoms, growth and adverse events could occur after month 6 if triggered by change in BMI (max of 16 additional assessments). After month 6, participants with declining BMI were re-randomized to one of three weight recovery treatments (WRTs) (29). The numbers of pills were recorded at each visit and parents completed a monthly log. For the participants who were rerandomized to WRTs, stimulant dose was capped until BMI restabilized at a healthy range.

### Measures

#### IOWA Conners Rating Scale

The IOWA measures disruptive, inattentive and dysregulated behavior in youth (40–42). Items were rated using Likert Scales that range from 0 (*Not at All*) to 3 (*Very Much*). The first IOWA was completed at baseline for all participants and were completed an average of 13 times over the course of the study (*M* 12.6, *SD* 3.2, range 1–18). At each time point, relevant items were averaged to compute the following scores: Inattentive-Overactive-Impulsive (*M* 1.38, *SD* 0.66, α 0.78), Oppositional-Defiant (*M* 0.93, *SD* 0.75, α 0.87), and Emotional Lability (*M* 0.89, *SD* 0.75, α 0.84). Emotional Lability score was computed as the average of the following items: “temper outburst” “demands must be met immediately” “cries often and easily” and “mood changes quickly and drastically” (42, 43). Previous research supports the psychometric properties of these scores (42, 44).

#### Impairment Rating Scale

The Impairment Rating Scale (IRS) measures impairment in peer relationships, getting along with parents, academic performance, classroom behavior, self-esteem, functioning in the family, and overall^1^ (45). Items were scored using a 0 to 6 metric, with higher scores indicating greater impairment. Parents completed the IRS an average of 13 times over the course of the study (*M* 12.6, *SD* 3.2, range 1–18). As in past studies, each item on the IRS was examined as an outcome (43, 46). The test-retest and inter-rater reliability and criterion validity of the IRS items have been supported in several samples with correlations ranged from 0.40 to 0.80 (45, 47, 48).

#### Pittsburgh Side Effects Rating Scale

The Pittsburgh Side Effects Rating Scale (PSERS) measures side effects that are often associated with medication treatment of ADHD (49). Items were rated using Likert Scales that ranged from 0 (*None*) to 3 (*Severe*). Parents completed the PSERS an average of 12 times over the course of the study (*M* 12.3, *SD* 3.3, range 1–18) with the first always at baseline. At each time point items were averaged to compute a total side effect score (*M* 0.40, *SD* 0.33, α 0.75) and items assessing mood-related side effects (Worried/anxious; Dull, tired, listless; Crabby, irritable; Tearful, sad, depressed; Socially withdrawn; Trouble Sleeping) were averaged to complete an Emotional Side Effects score (*M* 0.46, *SD* 0.46, α 0.74)^2^. Previous research supports the psychometric properties of these scores (8, 18).

### Analytic Plan

Groups (IRR vs. Non-IRR) were compared on baseline data using one-way ANOVAs or chi-square tests. Time and treatment effects were examined using mixed models computed in Proc Mixed in SAS 9.4. Medication (Med), Time, IRR, and the interactions between them were predictors, along with Behavior Therapy as a covariate. IRR was a categorical variable (Non-IRR = 0, IRR = 1). Medication (percent of study days medicated), Time (months of study, with zero as baseline), and Behavior Therapy (number of sessions) were continuous measures. A random intercept term was included to accommodate non-dependence due to repeated measures. Variance explained by fixed effects was estimated by computing marginal *R*^2^-values and variance explained by fixed and random effects was estimated by computing conditional R^2^-values (50). Significant interactions were probed by computing simple slopes of time separately by Group at lower vs. higher medication use. Lower medication use was defined as never taking medication during the study and higher medication use was defined as taking medication 70% of study days which was approximately 1 standard deviation above the sample mean and equates to using medication every weekday. Average daily dose is in equivalents of MPH and amphetamine doses were converted to MPH using the formula from the MTA (51). Mixed models were estimated using restricted maximum likelihood and Kenward-Roger adjusted degrees of freedom, as recommended for modest sample sizes (52). Consistent with the focus of the paper, only significant effects involving IRR were discussed.

## Results

### Group Differences at Baseline

Table 1 and Supplementary Table summarize demographic and rating scale characteristics of the full sample and separately for the IRR and Non-IRR groups. As shown, the IRR group had significantly more severe ADHD and ODD symptoms and more impairment in every area except academics. Side effects ratings prior to study treatment were also significantly higher in the IRR vs. Non-IRR group. Groups did not differ on demographic or treatment variables including medication dose.

**Table 1:** Demographic, treatment, and rating scale values as a function of baseline irritability (IRR).

	**Full sample**		**Non-IRR**	**IRR**	**Statistical test**		**Effect size**
	**(*****n*** **=** **226)**		**(*n* = 163)**	**(*n* = 63)**			
	***N***	**%**	**%**	**%**	***χ^2^***	***p***	***OR***
Male	164	72.9%	71.2%	77.4%	0.9	0.346	1.42
Race/Ethnicity					0.47	0.925	
Non-white non-hispanic	25	11.1%	10.4%	12.9%			1.25
Non-white hispanic	9	4.0%	3.7%	4.8%			1.31
White non-hispanic	37	16.4%	16.6%	16.1%			0.95
White hispanic	154	68.4%	69.3%	66.1%			0.88
Medicated during study	161	71.2%	71.2%	71.4%	0.00	0.969	1.01
	***M***	***SD***	***M***	***M***	***F***	***p***	***SMD***
Age	7.6	1.96	7.6	7.5	0.13	0.717	−0.05
Mean DBD rating^a^							
Inattention	2.0	0.6	1.9	2.2	10.8	0.001	0.48
Hyper-Imp	1.7	0.7	1.5	2.1	33.5	0.000	0.79
ODD	0.9	0.7	0.6	1.7	214.4	0.000	1.57
Non-irritable ODD	0.9	0.7	0.7	1.5	98.7	0.000	1.23
Irritable ODD	0.9	0.8	0.6	1.9	327.9	0.000	1.70
IOWA conners							
Inattent-overact-impulse	1.9	0.6	1.8	2.2	18.1	0.000	0.61
Oppositional-defiant	1.2	0.8	0.9	1.9	97.6	0.000	1.22
Emotional lability	1.2	0.8	0.9	2.0	117.4	0.000	1.31
Impairment rating scale							
Peer relationships	3.0	1.9	2.6	4.2	36.1	0.000	0.83
Parent relationships	3.6	1.8	3.3	4.4	20.9	0.000	0.65
Academic performance	4.5	2.0	4.4	4.8	1.5	0.231	0.18
Self esteem	3.7	1.8	3.4	4.5	18.7	0.000	0.61
Functioning in family	3.9	1.6	3.7	4.5	12.2	0.001	0.51
Overall impairment	4.8	1.2	4.6	5.1	8.4	0.004	0.43
PSERS^b^							
Total side effects	0.4	0.3	0.3	0.5	21.2	0.000	0.66
Mood side effects	0.5	0.5	0.4	0.7	26.0	0.000	0.73
Months in study	26.7	11.0	25.9	25.1	0.2	0.619	−0.07
Behavior therapy sessions	7.5	7.1	7.3	8.2	0.7	0.401	0.13
Average daily dose^c^	23.4	6.9	23.0	24.3	1.2	0.280	0.19
Percentage of study days on Med							
All participants	34.8	30.0	33.7	37.8	0.8	0.357	0.14
Medicated only (*n* = 161)	48.9	24.0	47.4	52.9	1.7	0.186	0.23

*M/N, mean for continuous variables or sample size for categorical variables; IRR, irritable; Percentage of study days on med is the percentage of study days that participants took medication, including participants who never took medication; F/χ^2^, f-ratio or chi-square value from comparisons of IRR groups (no vs. yes); OR, Odds Ratio; SMD, Standardized mean difference computed as: (M_irr_ – M_nonirr_)/SD_fullsample_; Medicated during study is number of participants who were medicated during at least part of the study*.

a *Disruptive behavior disorders rating scale*.

b *Pittsburgh side effects rating scale*.

c *MPH (mg/day)*.

### Group Differences Over Time and Treatment

#### IOWA Conners

##### Inattentive-Overactive-Impulsive

There were significant main effects of Time, Medication, and IRR. The main effect of IRR showed that higher Inattentive-Overactive-Impulsive (IO) scores were associated with being in the IRR group (Table 2).

**Table 2:** Parameter estimates for fixed effects in mixed model analyses.

***Measure***	***Intcpt***	***Bmod***	***Time***	***Med***	***IRR***	Time^*^Med	Time^*^IRR	Med^*^IRR	T^*^M^*^IRR	***R^**2**^m***	***R^**2**^c***
IOWA conners^a^											
Inat Imp Overact	1.53^*^	0.005	−0.009^*^	−0.303^*^	0.300^*^	−0.001	0.000	0.012	−0.011	0.18	0.53
Oppose Defiant	0.93^*^	0.001	−0.007^*^	−0.283	0.808^*^	0.007	0.009^*^	−0.053	−0.038^*^	0.21	0.68
Emotion Lability	0.87^*^	−0.001	−0.008^*^	−0.057	0.934^*^	−0.002	0.001	−0.274	−0.025^*^	0.21	0.64
Impairment^b^											
Peer relationships	2.22^*^	0.017	−0.010	−0.008	1.535^*^	0.019	−0.011	−0.519	−0.014	0.20	0.63
Relation w/ parent	3.03^*^	0.001	−0.013^*^	−0.411	0.946^*^	0.024^*^	0.001	0.248	−0.042^*^	0.20	0.62
Academics	4.42^*^	−0.020	−0.023^*^	−1.295^*^	0.002	0.033^*^	0.001	1.117	−0.041	0.18	0.49
Self esteem	3.17^*^	−0.009	−0.019^*^	−0.578	0.701^*^	0.020	0.004	0.890	−0.036	0.19	0.58
Function in family	3.21^*^	0.015	−0.024^*^	−0.563	0.612	0.027^*^	0.008	0.795	−0.048^*^	0.20	0.62
Overall impair	3.92^*^	−0.006	−0.024^*^	−0.255	0.556^*^	0.022^*^	0.006	0.443	−0.040^*^	0.18	0.50
PSERS^c^											
Total SE	0.36^*^	−0.006^*^	−0.001	0.113	0.163^*^	0.006^*^	0.003	−0.009	−0.012^*^	0.20	0.60
Emotional SE	0.42^*^	−0.008^*^	−0.000	0.119	0.292^*^	0.003	0.003	−0.077	−0.013^*^	0.20	0.60

Bmod, behavior modification main effect (# of sessions); Time, time main effect; Med, Medication main effect (% of study days medicated); IRR, Irritability main effect; Time^*^Med, Time by Medication interaction, Time^*^IRR, Time by Irritability interaction; Med^*^Irr, Medication by Irritability interaction; T^*^M^*^IRR, Time by Medication × Irritability interaction. Inat Imp Overact, mean score on the inattentive impulsive overactive scale; Oppose Defiant, mean score on the oppositional defiant scale; Emotion Lability, mean score on the emotional lability scale. Total SE, mean across all side effects items; Emotion SE, mean across emotion-related side effects. R^2^m, marginal R^2^-value, which estimates variance explained by the fixed effects. R^2^c, conditional R^2^-value, which estimates variance explained by fixed and random effects.

a *IOWA conners*.

b *Impairment rating scale*.

c *Pittsburgh side effects rating scale*.

* *p < 0.05*.

##### Oppositional-Defiant

**Figure 1:**
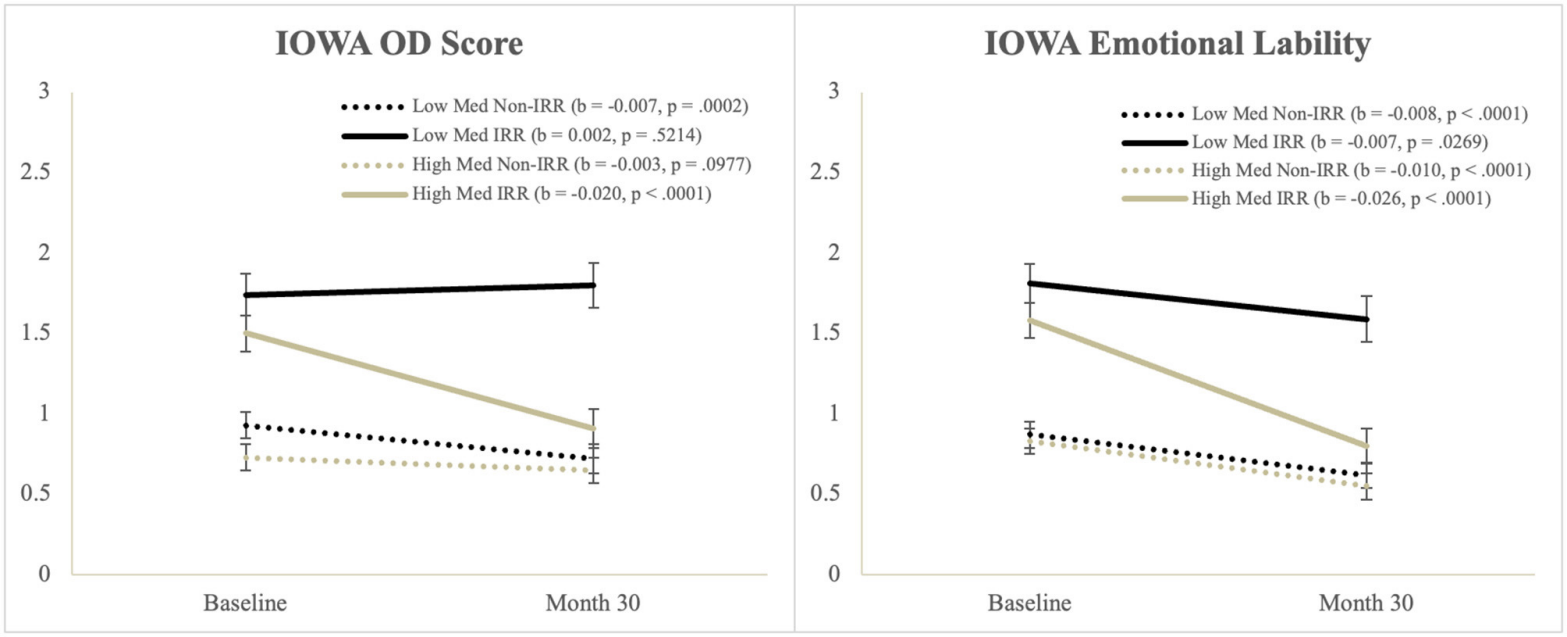
Illustration of the Time*Med*IRR interaction from the IOWA scale.

There were significant main effects of Time and IRR, and a significant Time^*^IRR interaction, but these were qualified by a significant Time^*^Medication^*^IRR interaction. Simple slopes tests of the three-way interaction (Figure 1) showed that Oppositional-Defiant (OD) scores significantly decreased for: (a) the IRR group with higher medication use and (b) the Non-IRR group who had lower medication use.

##### Emotional Lability

There were significant main effects of Time and IRR, but these were qualified by a significant Time^*^Med^*^IRR interaction. Simple slopes tests of the three-way interaction (Figure 1) showed that the IRR group with lower medication use had the least decrease in emotional lability scores.

#### Impairment

##### Peer Relationships

There was a significant main effect of IRR, which showed that the IRR group had higher peer impairment than the Non-IRR group (Table 2).

##### Relationship With Parents

**Figure 2:**
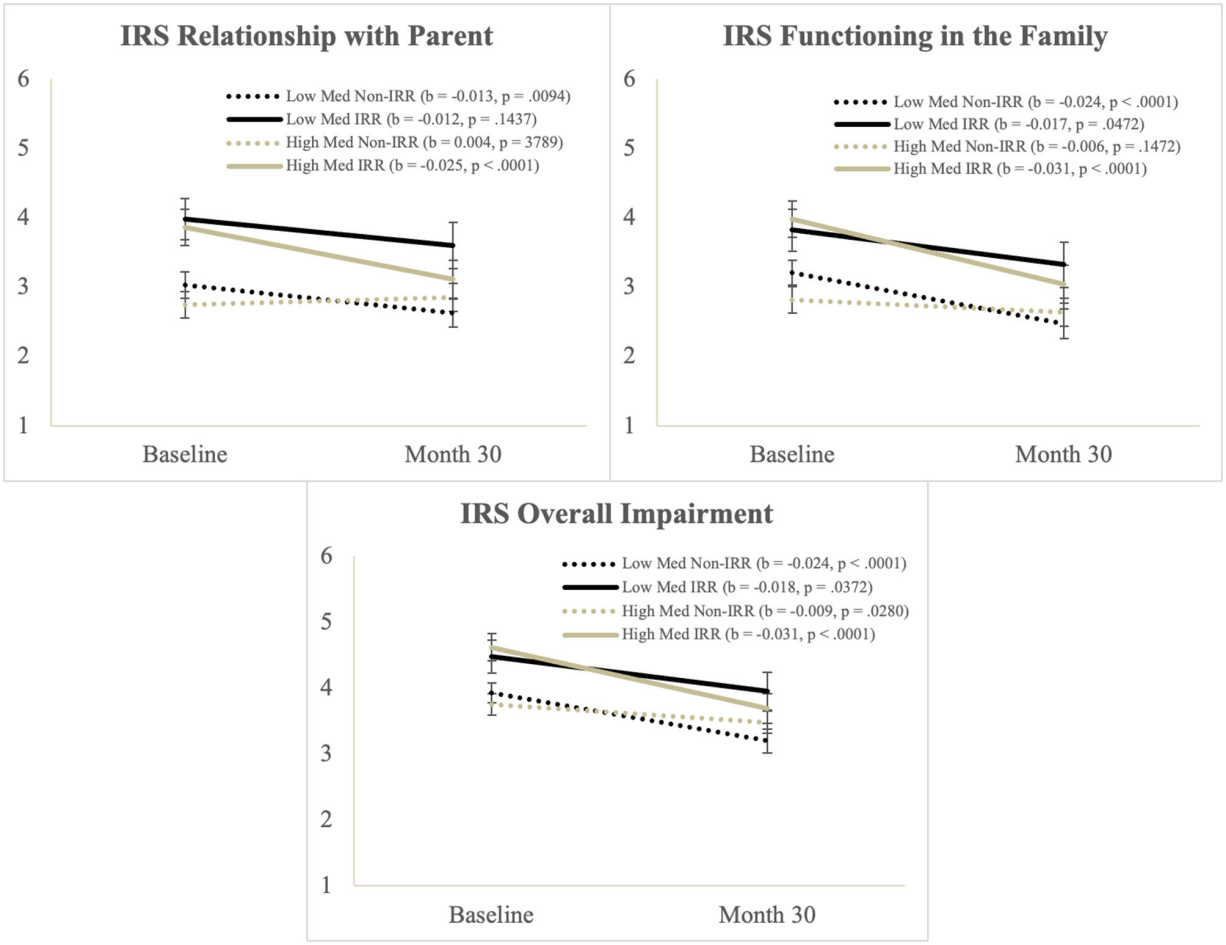
Illustration of the Time*Med*IRR interaction from the Score from the impairment rating scale.

There was a significant main effect Time and IRR, and a significant Time^*^Med interaction, but these were qualified by a significant Time^*^Med^*^IRR interaction. Simple slopes tests of the three-way interaction (Figure 2) showed that impaired relationships with parents decreased over time for the IRR group with higher medication use and for the Non-IRR group on lower medication use.

##### Academics

There were significant main effects of Time and Med, and a significant Time^*^Med interaction but no significant effects involving IRR.

##### Self-Esteem

There was a significant main effect of Time and IRR. The main effect of IRR showed that self-esteem impairment was higher in the IRR group than the non-IRR group.

##### Functioning in the Family

There was a significant main effect Time and a significant Time^*^Med interaction, but these were qualified by a significant Time^*^Med^*^IRR interaction. Simple slopes tests (Figure 2) showed that impaired functioning in the family decreased over time for youth except the Non-IRR group with higher medication use, who started off with the lowest levels of impairment on this measure.

##### Overall Impairment

There was a significant main effect Time and IRR and a significant Time^*^Med interaction, but these were qualified by a significant Time^*^Med^*^IRR interaction. Simple slopes tests (Figure 2) showed that overall impairment improved over time for all groups but the improvement was largest in the IRR group with higher medication use.

#### Pittsburgh Side Effects Rating Scale

##### Total Side Effects

There was a significant main effect IRR, and a significant Time^*^Med interaction, but these were qualified by a significant Time^*^Med^*^IRR interaction. Simple slopes tests showed that total side effects increased for Non-IRR participants with higher medication use but did not change for other participants (Table 2, Supplementary Figure).

##### Emotional Side Effects

There was a significant main effect of IRR, but this were qualified by a significant Time^*^Med^*^IRR interaction. Simple slopes tests showed that emotional side effects decreased for IRR participants with higher medication use but did not change for other participants.

## Discussion

This was the longest study and one of the largest studies to date to examine the impact of irritability on both the efficacy and tolerability of extended-release CNS stimulants in youth with ADHD. Study strengths include the size and diversity of the sample, the duration of treatment and methods for measuring medication use. Approximately 28% of participants had prominent irritability, which was associated with higher levels of ADHD symptoms, ODD symptoms, and impairment across multiple domains. Irritable youth also had higher rates of emotional lability as well as other physical and emotional symptoms at study entry and over the duration of the study. As hypothesized, significant improvements in levels of ADHD/ODD symptoms and impairment were seen. In youth with elevated levels of irritability, increasing medication use was associated with greater reductions in ODD symptoms, emotional lability, and impairment. We found little evidence that treatment worsened emotional lability. Results support the use of CNS stimulants as a first line treatment for children with ADHD and prominent irritability.

Similar rates of irritability in youth meeting criteria for ADHD have been reported elsewhere (23, 33). Even when using diagnostic criteria for DMDD instead of dimensional levels of irritability, nearly a third of ADHD youth will meet criteria (53). Across samples, irritability is associated with elevated levels of internalizing and externalizing symptoms (53). Impairment findings are consistent with prior reports documenting appreciably disturbed functioning across a wide range of domains in youth with persistent irritability compared to youth with ADHD without prominent irritability (1, 8). The combined results suggest that irritability is a potentially impactful treatment target for improving current functioning that could also reduce the risk of future comorbidity (54).

Across measures, participants with elevated irritability exhibited a degree of improvement that was comparable or more robust than those without elevated irritability. In this sample, that was not self-selected for irritability, medication reduced baseline group severity differences for ODD symptoms and for emotional lability which were assessed using different measures. These efficacy results are consistent with the MTA and with studies enrolling youth meeting criteria for DMDD or Severe Mood Dysregulation (6, 18, 22). Youth with ADHD, irritability and lower medication use were most likely to remain more symptomatic and appreciably impaired. In this group, no significant change was seen in emotional lability or ODD symptoms, and they had less improvement in family functioning and parent-child relationships. Ross and colleagues observed that aggression and symptoms of ODD are a common reason for increasing dose, although this study did not report on the benefits of these dose increases (9). Study procedures for dose optimization may differ from routine care as they were based on standardized reassessments every 2 weeks integrating parent and teacher report, which have been found to enhance outcomes (55, 56). We did observe greater improvements in youth with IRR using medication more frequently, suggesting the frequency of use may be as important as mean daily dose.

Changes in symptoms scores are often not well-correlated with reduced functional impairment making it essential to measure impairment directly (57, 58). While impairment rates did not decline to levels seen in non-irritable youth, irritable participants exhibited clinically meaningful gains in their relationship with parents, family functioning, and overall functioning. Acute reductions in impairment at home may translate into long-term enhancements of family functioning (59), whereas persistently elevated levels of family conflict may increase the risks of range of future psychopathology (59, 60). Even with treatment, youth with elevated levels of irritability continued to experience more social impairment than non-irritable youth. The limited impact of medication on the social functioning of youth with ADHD is consistent with previously published work and suggests the need for multimodal interventions (61).

There was no evidence of worse medication tolerability in irritable vs. non-irritable youth in this large sample of treatment naïve youth. Non-emotional side effects were more strongly correlated with medication use than irritability status. When emotional symptoms were elevated at baseline, they decreased only in those treated with medication. In a 4-week trial of youth with predominantly the inattentive presentation of ADHD, diminishing irritability, other internalizing symptoms and physical symptoms were observed in those participants with elevated ODD symptoms at baseline subsequently treated with CNS stimulants. The opposite pattern was seen in those with low entry levels of irritability and other ODD symptoms (27). Our results extend these findings over a two- and half-year duration and to children with elevated levels of impulsivity/hyperactivity as well as inattention.

We found little evidence that stimulants need to be more cautiously titrated in youth with irritability. Pretreatment assessment of emotional and physical health followed by frequent measurement of side effects, symptoms, and impairment using standardized measures allow for more precise assessments of treatment effects that may improve the ability to identify if observed irritability is related to medication use, ADHD itself or other environmental triggers. Failure to collect structured, pretreatment ratings of irritability can lead to misclassifying treatment related improvements as medication-induced adverse events.

It is important to note that this sample was not recruited based on levels of irritability. In samples selected for prominent levels of irritability or aggression, significant improvements in aggression and other behavioral problems are also seen to the degree that a sizeable subset of youth do not need additional treatments beyond CNS stimulants and a relatively low intensity behavioral therapy (17–19, 22). Blader and colleagues found that optimization of the ADHD regiment avoided the use of adjunctive antipsychotics in nearly two-thirds of youth with ADHD and persistent aggression (19). However, a meaningful subset of youth continues to exhibit impairing levels of dangerous behaviors. In cases where marked impairment persists after systematic dose optimization of CNS stimulants, there is evidence that treatment with antidepressants, mood stabilizers and atypical antipsychotics leads to incremental improvement (17, 19, 20). However, outside of clinical trials, these adjunctive agents are often used before the CNS stimulant regiment is optimized (62).

This study is unique as the majority of participants were Hispanic who have been largely underrepresented in previous studies (17, 19, 20, 55). We observed no impact of ethnicity on treatment results, suggesting that findings are generalizable to non-Hispanic youth (63). Another strength of the study is its duration. The duration of most prior treatment studies examining irritability was only weeks while the MTA analyzed the impact of irritability over 14 months. In this sample, effects of CNS stimulants were assessed over 30 months, spanning 3 school years for some participants. All ADHD medication was provided through the study allowing for precise assessments of medication at least every 3 months by direct pill count and review of medication logs.

## Limitations

The findings of the present study should be considered within the context of several limitations. A main limitation of this study is the lack of a gold standard definition of irritability, which has led to a variety of descriptive and diagnostic terms being used to label children with frequent temper outbursts and high amounts of negative affect (64). There is appreciable evidence for a bifactor model of ODD with distinct irritability and defiance dimensions (65–68). Therefore, we used the 3 ODD irritability items to define diagnostic group status similar to analyses employed in the MTA and other studies (65). The term emotional lability has long been applied to ADHD youth in recognition that they may struggle to regulate both negative and positive emotions. It has been found to be dimensionally distinct from irritability with the two having differential associations with other internalizing and externalizing symptoms (25, 69). Therefore, we elected to employ emotional lability as the primary metric of treatment effects rather than relying on the same irritability definition used to define group membership. When we removed the one overlapping item of “temper outbursts” between our irritability and emotional lability measures, results were unchanged. Irritability appears to manifest in both phasic and tonic patterns. Phasic irritability has been found to more strongly associated with ADHD (70) and to be more responsive to pharmacological treatment (20). Unfortunately, we were not able to separate these two patterns of irritability with the measures used in this study but that should be a focus of future work.

In addition to the limitations regarding the assessment of irritability, this study was conducted as a secondary analysis of a larger study examining growth patterns in children with ADHD. There was not a randomly assigned no-treatment group limiting the capacity to interpret change over time. Medication treatments were not blinded, so expectancy bias may have inflated observed treatment effects. A prior meta-analysis found differential impacts of amphetamine vs. MPH products for irritability (71). There were not sufficient children prescribed amphetamine to assess the impact of stimulant class in this sample. Teacher ratings were collected but the study spanned up to 3 school years so there was appreciable inter-rater variability leading us to preferentially focus on parent ratings. There is a need for future research to test if the same pattern extends to teacher-defined irritability. Finally, it is possible that the trial design rules for medication to promote physical growth may have impacted irritability results. For example, 20% of participants were initially randomized to a no medication group for the first 6 months and dose could not be increased once assigned to a weight recovery arm.

## Conclusions

Attention deficit hyperactivity disorder youth with elevated levels of irritability manifest higher levels of ADHD symptoms, other problem behaviors and impairment. The use of CNS stimulants was associated with improvements in all of these realms, with the largest treatment effects seen in irritable youth who regularly took medication. Regular medication treatment significantly reduced baseline differences between irritable and non-irritable participants for symptoms of ADHD, emotional lability, other symptoms of ODD, and impairment. Youth with ADHD and irritability tolerated CNS stimulants equally well as youth without irritability. Given these results, it appears reasonable to consider CNS stimulants as part of the initial treatment package for youth with ADHD and elevated levels of irritability.

## Data Availability Statement

The raw data supporting the conclusions of this article will be made available by the authors as per National Institute of Health policy at the time of funding, without undue reservation.

## Ethics Statement

The studies involving human participants were reviewed and approved by Western Institutional Review Board. Written informed consent to participate in this study was obtained from parents and assent was obtained from children age seven or older.

## Author Contributions

RB: conceptualization, methodology, writing—original draft, review, and editing. DW: conceptualization, methodology, formal analysis, writing—original draft, review, and editing. WP (3rd author): conceptualization, methodology, and writing—review and editing. WP (4th author): conceptualization, methodology, data collection, writing—review and editing, supervision, project administration, and funding acquisition. JW: conceptualization, methodology, investigation, resources, data collection, writing—original draft, review, editing, supervision, project administration, and funding acquisition. All authors contributed to the article and approved the submitted version.

## Conflict of Interest

JW has received research funding from the National Institutes of Health, Supernus, and Pfizer in the past 3 years. WP (4th author) has received funding from NIMH, NIAAA, NIDA, and the Institute of Education Sciences. The remaining authors declare that the research was conducted in the absence of any commercial or financial relationships that could be construed as a potential conflict of interest.

## Footnotes

^1^An item assessing impairment was siblings was also administered but is not used because not all children had siblings.

^2^A score computed using non-mood side effect items was low, suggesting they rarely occurred, and had poor reliability (*M* 0.34, *SD* 0.31, α 0.52). As such it was not used in the study.
