# Supplementary material for: The Impact of Persistent Irritability on the Medication Treatment of Paediatric Attention Deficit Hyperactivity Disorder
Source: Front Psychiatry. 2021 Jul 21;12:699687. doi: 10.3389/fpsyt.2021.699687 (PMC8333707; doi:10.3389/fpsyt.2021.699687)
Supplement: Supplementary file 1 [file Table_1.DOCX]

Supplement Table

*Means, standard deviations and effect sizes for rating scales collected at dose optimization and at the end of the study.*

|  | Dose Optimization | | | | End of Study | | | | Effect Sizes | |  |
| --- | --- | --- | --- | --- | --- | --- | --- | --- | --- | --- | --- |
|  | Full Sample | | Non-IRR | IRR | Full Sample | | Non-IRR | IRR | Dose | End |  |
|  | M | SD | M | M | M | SD | M | M |  |  | |
| Mean DBD rating^a^ |  |  |  |  |  |  |  |  |  |  | |
| Inattention | -- | -- | -- | -- | 1.4 | 0.7 | 1.4 | 1.6 | -- | 0.28 | |
| Hyper-Imp | -- | -- | -- | -- | 1.1 | 0.7 | 1.0 | 1.5 | -- | 0.64 | |
| ODD |  |  |  |  |  |  |  |  |  |  | |
| Non-irritable ODD | -- | -- | -- | -- | 0.7 | 0.6 | 0.6 | 1.2 | -- | 0.94 | |
| Irritable ODD | -- | -- | -- | -- | 0.7 | 0.7 | 0.5 | 1.3 | -- | 1.13 | |
| IOWA Conners |  |  |  |  |  |  |  |  |  |  | |
| Inattent-Overact-Impulse | 1.2 | 0.6 | 1.1 | 1.5 | 1.3 | 0.6 | 1.3 | 1.5 | 0.57 | 0.28 | |
| Oppositional-Defiant | 0.8 | 0.7 | 0.6 | 1.3 | 1.0 | 0.8 | 0.8 | 1.4 | 0.90 | 0.78 | |
| Emotional Lability | 0.9 | 0.7 | 0.7 | 1.4 | 0.8 | 0.8 | 0.7 | 1.3 | 1.00 | 0.82 | |
| Impairment Rating Scale |  |  |  |  |  |  |  |  |  |  | |
| Peer relationships | 2.3 | 1.7 | 2.0 | 3.1 | 2.7 | 1.8 | 2.4 | 3.4 | 0.63 | 0.57 | |
| Parent relationships | 2.7 | 1.6 | 2.4 | 3.4 | 3.0 | 1.8 | 2.8 | 3.5 | 0.60 | 0.38 | |
| Academics | 3.3 | 1.9 | 3.1 | 3.6 | 3.8 | 1.7 | 3.8 | 3.9 | 0.25 | 0.05 | |
| Self esteem | 2.7 | 1.7 | 2.4 | 3.4 | 3.1 | 1.7 | 2.8 | 3.7 | 0.61 | 0.47 | |
| Functioning in family | 2.8 | 1.8 | 2.5 | 3.6 | 3.1 | 1.8 | 3.0 | 3.5 | 0.63 | 0.31 | |
| Overall impairment | 3.6 | 1.6 | 3.4 | 4.0 | 3.7 | 1.5 | 3.6 | 4.1 | 0.36 | 0.30 | |
| PSERS^b^ |  |  |  |  |  |  |  |  |  |  | |
| Total side effects | 0.4 | 0.3 | 0.4 | 0.5 | 0.4 | 0.3 | 0.4 | 0.5 | 0.36 | 0.24 | |
| Mood side effects | 0.5 | 0.5 | 0.5 | 0.6 | 0.5 | 0.4 | 0.4 | 0.6 | 0.37 | 0.33 | |

**Notes**: Sample size for the dose optimization data was 141 (Non-irr = 101, Irr = 39) it excludes children who were never medicated during the study. The DBD ratings are only presented for end of study because they were not collected (and therefore not available) as part of dose optimization. Effect sizes are standardized mean differences comparing the irritable and non-irritable groups at dose optimization (Dose) and at end of study (End) using the following formula: (M_irr_ – M_nonirr_) / SD_fullsample._  IRR = irritable. a = Disruptive Behavior Disorders Rating Scale. b = Pittsburgh Side Effects Rating Scale.
