# Supplementary figures and images for: The Impact of Persistent Irritability on the Medication Treatment of Paediatric Attention Deficit Hyperactivity Disorder
Source: Front Psychiatry. 2021 Jul 21;12:699687. doi: 10.3389/fpsyt.2021.699687 (PMC8333707; doi:10.3389/fpsyt.2021.699687)

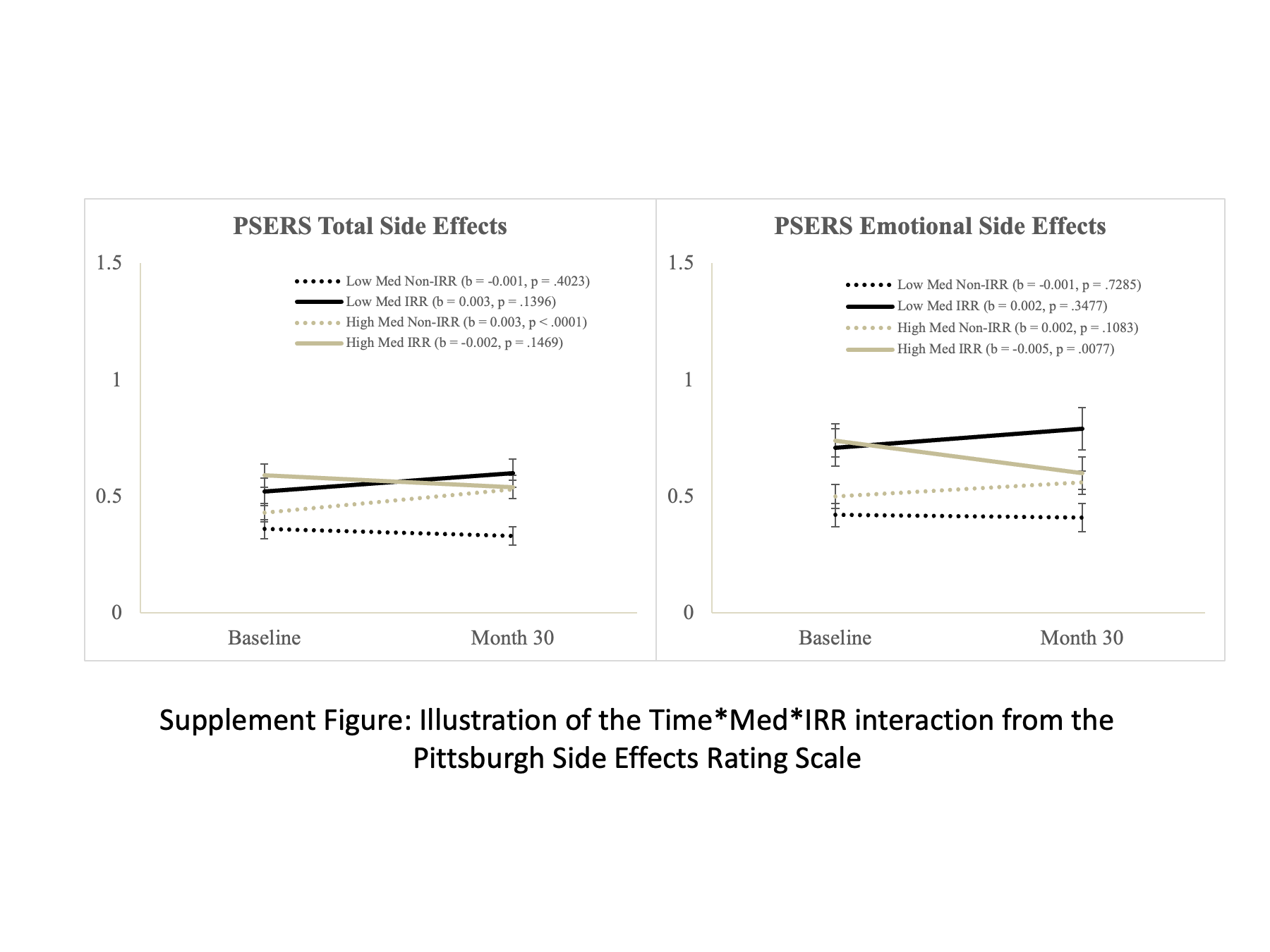

Supplement: Supplementary file 2 [file Image_1.TIFF]
